# Functional Characterization of the *mazEF* Toxin-Antitoxin System in the Pathogenic Bacterium *Agrobacterium tumefaciens*

**DOI:** 10.3390/microorganisms9051107

**Published:** 2021-05-20

**Authors:** Wonho Choi, Yoshihiro Yamaguchi, Ji-Young Park, Sang-Hyun Park, Hyeok-Won Lee, Byung-Kwan Lim, Michael Otto, Masayori Inouye, Min-Ho Yoon, Jung-Ho Park

**Affiliations:** 1 Bio-Evaluation Center, Korea Research Institute of Bioscience and Biotechnology, Cheongju, Chungbuk 28116, Korea; wonho.choi@nih.gov (W.C.); pjy125@kribb.re.kr (J.-Y.P.); 2 Pathogen Molecular Genetics Section, Laboratory of Bacteriology, National Institute of Allergy and Infectious Diseases, U.S. National Institutes of Health, Bethesda, MD 20814, USA; Michael.otto@nih.gov; 3 Graduate School of Science, Osaka City University, Sugimoto, Sumiyoshi-ku, Osaka 558-8585, Japan; yoshi@sci.osaka-cu.ac.jp; 4 Biotechnology Process Engineering Center, Korea Research Institute of Bioscience and Biotechnology (KRIBB), Cheongju, Chungbuk 28116, Korea; bakssang@kribb.re.kr (S.-H.P.); tntn7616@kribb.re.kr (H.-W.L.); 5 Department of Bioprocess Engineering, University of Science and Technology (UST) of Korea, 217 Gajeong-ro, Yuseong-gu, Daejeon 34113, Korea; 6 Department of Biomedical Science, Jungwon University, Goesan-gun, Chungbuk 28024, Korea; bklim@jwu.ac.kr; 7 Department of Biochemistry, Center for Advanced Medicine and Biotechnology, Rutgers-Robert Wood Johnson Medical School, Piscataway, NJ 08854, USA; inouye@cabm.rutgers.edu; 8 Department of Bio-Environmental Chemistry, College of Agriculture and Life Sciences, Chungnam National University, Daejeon 34134, Korea

**Keywords:** *Agrobacterium tumefaciens*, TA system, *mazF*, *MazE*, mRNA endonuclease

## Abstract

*Agrobacterium tumefaciens* is a pathogen of various plants which transfers its own DNA (T-DNA) to the host plants. It is used for producing genetically modified plants with this ability. To control T-DNA transfer to the right place, toxin-antitoxin (TA) systems of *A. tumefaciens* were used to control the target site of transfer without any unintentional targeting. Here, we describe a toxin-antitoxin system, *Atu0939 (mazE-at)* and *Atu0940 (mazF-at)*, in the chromosome of *Agrobacterium tumefaciens*. The toxin in the TA system has 33.3% identity and 45.5% similarity with MazF in *Escherichia coli*. The expression of MazF-at caused cell growth inhibition, while cells with MazF-at co-expressed with MazE-at grew normally. In vivo and in vitro assays revealed that MazF-at inhibited protein synthesis by decreasing the cellular mRNA stability. Moreover, the catalytic residue of MazF-at was determined to be the 24th glutamic acid using site-directed mutagenesis. From the results, we concluded that MazF-at is a type II toxin-antitoxin system and a ribosome-independent endoribonuclease. Here, we characterized a TA system in *A. tumefaciens* whose understanding might help to find its physiological function and to develop further applications.

## 1. Introduction

Many toxin-antitoxin (TA) systems are present on plasmids or chromosomes and consist of two small genetic elements, namely toxin and antitoxin [1]. The TA system is known to regulate cell growth or the arrest of bacteria for maintaining life under stressful environmental conditions, but its various proposed functions are still under debate, and its role is still unclear [1]. The TA systems were first identified in the plasmid F as *ccdAB* and in the plasmid R1 as *hok-sok* and *kis-kid* [2,3,4]. Among the type II TA systems on plasmid, the CcdAB TA system consisting of antitoxin CcdA and toxin CcdB was identified in the *Escherichia* *coli* F plasmid. CcdB inhibits DNA replication through the interaction with the DNA gyrase and leads to cell growth arrest, while the antitoxin CcdA neutralizes the CcdB toxin [5].

Recently, various TA systems were identified in bacterial chromosomes and classified into six different groups, from type I to VI, on the basis of the construction of antitoxins and the mode of action of the antitoxin to prevent the action of the toxin [6]. Among the TA system types, the type II TA system has been well studied for its mechanisms or physiological properties. Generally, type II TA systems consist of two proteins, a toxin and its cognate unstable antitoxin. The toxins in TA systems inhibit cell growth through cellular toxicity; in contrast, antitoxins neutralize the toxin activity [6]. Currently, 12 and 76 type II TA systems have been identified in *E. coli* K-12 and *Mycobacterium tuberculosis* H37Rv genomes, respectively. The *mazEF* TA system is classified as a type II TA system and has been identified on chromosomes from various bacterial species. The first *mazEF* TA system has been identified in the chromosome in *E. coli*, and its function has been reported to be programmed cell death in *E. coli* [7].

Moreover, toxins in type II TA systems not only inhibit DNA, RNA, or protein synthesis but are also involved in antibiotic resistance, biofilm formation, and persistent cell formation in the plasmid-based systems [5,8]. Some toxins in the TA systems target and cleave mRNA and are called mRNA interferases [9]. mRNA interferases are classified into ribosome-dependent or -independent toxins based on the ribosome dependency for their endoribonuclease activity [10]. MazF homologs are well known as ribosome-independent toxins and have sequence-specific endoribonucleases that can cleave mRNA sequences in a ribosome-independent manner [11]. RelE homologs have been reported to be ribosome-dependent and have an endoribonuclease activity under the interaction with bacterial ribosomes [12,13,14].

*Agrobacterium tumefaciens* is a gram-negative soil bacterium, and it is a serious plant pathogen. Recently, six candidates of TA modules or toxins (Atu0939-Atu0940, Atu1004, Atu2140, Atu3013, Atu6122, Atu6083) were identified using in an silico analysis of *A. tumefaciens* C58 [15]. Five of those six candidates showed cell growth inhibition, when overexpressed in *A. tumefaciens* gv3101 strain. *A. tumefaciens* is used for making genetically modified plants, because of the ability to transfer T-DNA of its Ti plasmid to host plants [15]. For this application, it is of important to control the target site of transfer without any unintentional targeting. A previous study showed the possibility of *pemIK*(Atu0939-Atu0940) to control the target site of T-DNA transfer. Moreover, *ietA/iteS* and *yoeB/yefM* TA systems have been identified on plasmids or chromosomes in *A. tumefaciens* [16,17]. Notably, *A. tumefaciens* YoeB toxin (AtYoeB) showed ribosome-independent non-specific nuclease activity cleaving both RNA and DNA, even though *E. coli* YoeB is a well-known RelE superfamily ribosome-dependent toxin [18,19]. In this study, we identified a novel TA system, MazF-at and MazE-at, in *A. tumefaciens*. MazF-at has endoribonuclease activity, and its cognate antitoxin MazE-at can neutralize the toxicity of MazF-at.

## 2. Materials and Methods

### 2.1. Bacterial Strains, Plasmids, Primers, and Chemicals

All the bacterial strains and plasmids used in this study are listed in Table 1. The *A. tumefaciens* EHA105 strain was grown in YEP (10 g yeast extract (BD, Sparks, MD, USA), 10 g Bacto peptone (BD, Sparks, MD, USA), and 5 g NaCl/L, pH 7.0 with spectinomycin 100 µg/mL (Duchefa Biochemie, Shanghai, China)) at 28 °C in a shaker incubator (180 rpm). DH5α, BW25113, and BL21(DE3) of *E. coli* strains were used for cloning, cell toxicity, and protein expression assays. pBAD24, pET28a (BBI Life Sciences, Novagen, Darmstadt, Germany), and pGEX 6p-1 (Merck, St. Louis, MO, USA) plasmids were used in this study. The antibiotics for *E. coli* ampicillin (Duchefa Biochemie, Haarlem, Netherlands) and kanamycin (Merck, St. Louis, MO, USA) were used at concentrations of 100 µg/mL and 50 µg/mL, respectively.

### 2.2. Cloning of mazE, mazF, mazEF, and mazF Mutants

The *mazE-at*, *mazF-at*, and *mazEF-at* genes in the *mazEF*-at operon were separately amplified through PCR using *A. tumefaciens* gDNA as a template, and cloned into pBAD24, pET28a, and pGEX 6p-1 with specific primer sets (Table 2). The *mazF-at* mutants (MazF-at-E24N, -E24Q, -E24K, -E24D, and -E24A) were constructed by site-directed mutagenesis with the use of the mutagenic oligonucleotide primers (Table 2) using pBAD24- *mazF-at* as template. The PCR products were digested with *DpnI* to remove the template DNA and transformed into the *E. coli* DH5α strain. All point mutations were confirmed using DNA sequencing.

### 2.3. Cell Toxicity on Plates and in Liquid Medium

The pBAD24-*mazE-at*, *mazF-at*, –*mazEF-at*, *and -mazF-at* mutants (MazF-E24N, -E24Q, -E24K, -E24D, and -E24A) were transformed into *E. coli* BW25113. To conduct the cell toxicity assay, transformed cells were grown on M9 agar plates with or without 0.2% L-arabinose (Merck, St. Louis, MO, USA) at 37 °C for 20 h. To construct the growth curve, transformed cells were grown in an M9 liquid medium with 0.5% glycerol at 37 °C in the presence or absence of 0.2% L-arabinose. The cell cultures were sampled at 0, 30, 60, 90, 120, 150, and 240 min after the induction.

### 2.4. Cellular Target Assay of DNA, RNA, and Protein Synthesis In Vivo

The pBAD24-mazF-at was transformed into *E. coli* BW25113. The transformed cells were inoculated in M9 medium with 0.5% glycerol and cultured at 37 °C. The culture was grown until the OD_600nm_ of the culture reached 0.4, and then MazF-at was overexpressed by induction with arabinose to a final concentration of 0.2%. Each 0.4 mL cell culture was sampled at 0, 5, 10, 30, and 60 min after the induction. Each sample was mixed with 10 µCi of [^3^H]-thymidine, 10 µCi of [^3^H]-uridine, or 30 µCi of [^35^S]-methionine, and 30 µg of nonradioactive thymidine, 30 µg of nonradioactive uridine, or 80 µg of nonradioactive methionine, respectively, and incubated at 37 °C for 30 sec. The rates of DNA, RNA, and protein synthesis were analyzed as previously described [19].

### 2.5. Protein Expression and Purification

To purify the N-terminal His_6_-tagged MazE-at, MazF-at, and MazEF-at complex, *E. coli* BL21(DE3) harboring pET28a-*mazE-at*, -*mazF-at*, or *–mazEF-at* was grown in LB medium at 37°C. The culture was grown until the OD_600nm_ of the culture reached 0.5, and then MazE-at, MazF-at, or MazEF-at was overexpressed by the induction with isopropyl-β-d-1-thiogalactoside (IPTG) (Merck, St. Louis, MO, USA) to a final concentration of 0.5 mM. After the induction, the cells were grown for 6 h at 18°C. The cultured cells were harvested by centrifugation (4392× *g* for 30 min at 4°C) and disrupted using a French pressure cell press. After the centrifugation (12,000× *g* for 30 min at 4 °C), soluble fractions were applied to Ni-NTA agarose (Qiagen, Hilden, Germany) following the manufacturer’s protocol. The purified proteins were applied to 15% sodium dodecyl sulfate-polyacrylamide gel electrophoresis (SDS-PAGE) and transferred to a polyvinylidene difluoride membrane (PVDF), and a Western Blot analysis was performed using the polyclonal anti-histidine tag antibody (#2365) (cell signaling Technology, Danvers, MA, USA).

### 2.6. mRNA Stability Analysis Using Northern Blot

The pBAD24-mazF-at was transfected into *E. coli* BW25113 cells. The transformed cells were cultured in M9 medium with 0.5% glycerol at 37 °C. The culture was grown until the OD_600nm_ of the culture reached 0.4, then MazF-at was overexpressed by induction with arabinose to a final concentration of 0.2%. Non-induced samples were prepared without the induction by arabinose in the same growth conditions as the induced samples. Aliquots of the cell cultures were collected at 0, 5, 10, 30, and 60 min after the induction. Total RNA was extracted by TRIzol (Thermo Fisher Scientific, Waltham, MA, USA) using the manufacturer’s protocol. The 30 µg of total RNA was applied to each lane onto denatured agarose gel. Northern blot was performed as previously described, with the modification that the probes were labeled with biotin-14-dCTP [20].

### 2.7. Endoribonuclease Activity Assay of MazE-at, MazF-at, and MazEF-at In Vitro

The 0.5 μM MazF-at, MazEF-at, or MazF-ec(E24A) protein was incubated with 0.8 μg/µL MS2 phage RNA (Roche Applied Science, Penzberg, Germany) in 20 mM Tris-HCl (pH 8.0) including 0.5 μL of RNA inhibitor (Roche Applied Science, Penzberg, Germany) at 37°C. The reactions were sampled at the indicated time intervals and were stopped by adding RNA loading dye (95% formamide, 0.025% SDS, 0.025% bromophenol blue, 0.025% xylene cyanol FF, 0.5 mM EDTA), and then the reactions were incubated for 3 min at 70 °C and electrophoresed on a 1.5% agarose gel.

### 2.8. Secondary Structure Analysis by Circular Dichroism

Far-UV CD measurements were obtained using an automated Chirascan CD spectrometer (Applied Photophysics Ltd., Leatherhead, UK). The far-UV spectra were determined over a wavelength range of 190–260 nm using 0.5-mm path length cell at 25 °C. All experiments were performed in 20 mM Tris HCl, pH 7.5, and then adjusted to a 0.25 mg/mL protein concentration of purified MazE-at and MazF-at for CD analysis. The background CD spectrum of the buffer was subtracted from each spectrum. The percentage of secondary structure content was calculated using the CDNN software [21].

### 2.9. Protein Interaction Analysis by GST Pull-Down Assay with Beads

The pGEX6p-1 (Glutathione S-transferase (GST) only), pGEX-6-1-*mazE-at* (GST-MazE-at), and pET28-*mazF-at* (6H-MazF-at) were transformed into *E. coli* BL21(DE3) and cultured in LB medium with 100 µg/mL ampicillin for pGEX6p-1 and 50 µg/mL kanamycin for pET28. When the OD_600nm_ of the cultures reached 0.5, 0.5 mM IPTG was added to induce the expression of GST, GST-MazE-at, and N-terminal His_6_-tagged MazF-at for 6 h at 18 °C. The cultured cells were harvested by centrifugation (4392× *g* for 30 min at 4 °C) and disrupted using a French pressure cell press. The cell debris were removed by centrifugation (12,000× *g* for 30 min at 4 °C), and soluble fractions were collected. GST only and GST-MazE-at were immobilized onto glutathione-Sepharose 4B beads by a gentle shaking motion on a rotating platform for 3 h at 4 °C, collected by centrifugation (1250× *g* for 1 min), and washed three times with PBS solution. The soluble fraction of MazF-at was incubated with GST beads by a gentle shaking motion on a rotating platform for 3 h at 4 °C and was collected by centrifugation (1250× *g* for 1 min). Beads were washed three times with PBS solution, separated by SDS-PAGE, and detected by Western Blotting with an anti-histidine antibody (#2365) (cell signaling Technology, Danvers, MA, USA).

## 3. Results

### 3.1. MazF-at Is Toxic in E. coli and Is Neutralized by MazE-at

**Figure 1:**
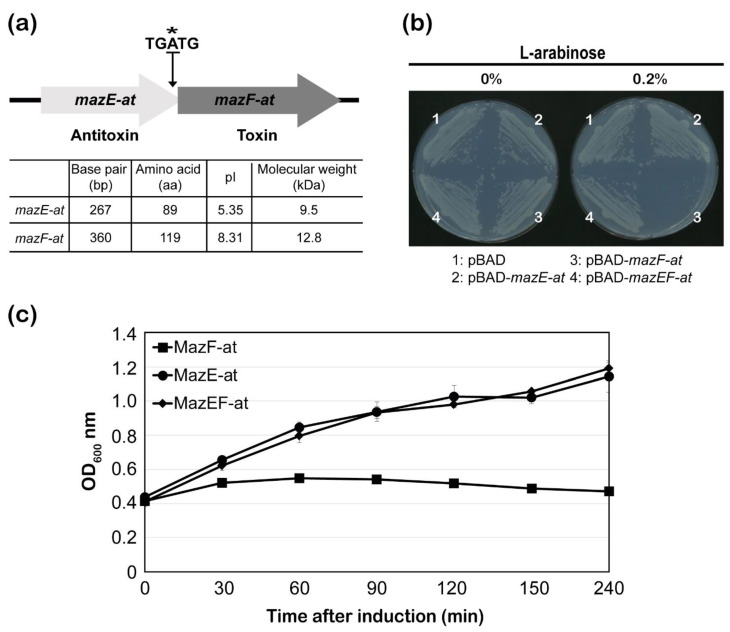
The characterization of the MazEF-at TA system in *Agrobacterium tumefaciens*. (**a**) Gene map of the *mazEF-a*. The asterisk indicates the nucleotide where the orf of the two genes overlap. The table includes the gene length, number of amino acids, theoretical pI, and molecular weight of MazE-at and MazF-at; (**b**) Toxicity of MazF-at on plates. The cells of *Escherichia* *coli* BW25113 harboring pBAD24-*mazE-at* (2), -*mazF-at* toxin (3), -*mazEF-at* (4) or pBAD24 only (1) were incubated on the M9 plate with or without 0.2% L-arabinose; (**c**) Effect of MazF-at on cell growth induction. The culture of *E. coli* BW25113 harboring pBAD24-*mazE-at* (black circle), -*mazF-at* (black square), or –*mazEF-at* (black rhombus) was grown until the OD _600nm_ (optical density) of the culture reached ~0.4, followed by adding 0.2% L-arabinose at 0 min, and bacterial growth was measured for 6 h at OD_600_ nm. Error bars represent the standard error of the mean (SEM).

Using the toxin-antitoxin database (TADB 2.0), we identified 13 candidates (Table S1) for TA systems in *A. tumefaciens* C58. We found that six clones (Atu0940, Atu2017, Atu0674, Atu0934, Atu1004, and Atu8169) out of 13 showed toxicity (Figure S1, Table S1) [22]. With this screening of TA systems, we also found that two genes, *mazE-at (Atu0939)* and *mazF-at (Atu0940)*, later classified as one of the *mazEF* homologs, had been predicted as a potential TA system in the chromosome of *A. tumefaciens*. The two genes, *mazE-at* and *mazF-at*, are located in the same operon, and the stop codon of *mazE-at* overlaps with the start codon of *mazF-at* by one nucleotide (Figure 1a, asterisk). The two proteins, MazE-at and MazF-at, consist of 89 and 119 amino acid residues, respectively, and the pI values are 5.35 and 8.31, respectively (Figure 1a). MazF-at (Atu0940) was identified as *pemIK* TA system in the previous study [15]. They found six candidates of TA modules in *A. tumefaciens* C58, and five of those six candidates showed growth inhibition in *A. tumefaciens* gv3101 strains [15]. In this study, we further evaluated whether the two genes, *mazE-at* and *mazF-at*, function as a TA system. *mazE-at*, *mazF-at*, and *mazEF-at* were amplified using genomic DNA extracted from *A. tumefaciens* and cloned into the pBAD24 vector. The plasmids pBAD24-*mazE-at*, *mazF-at*, and *mazEF-at* were transformed into *E. coli* BW25113 cells, and cell toxicity was tested on plates with or without 0.2 % L-arabinose. When MazE-at or MazEF-at were induced, transformant colonies were formed on the plate. However, when MazF-at was induced in the presence of 0.2 % arabinose, no colonies were formed. We also examined the effect of MazF-at inducing cell growth in a liquid culture (Figure 1b). As shown in Figure 1c, growth inhibition was immediately observed after the induction of MazF-at but not MazE-at or MazEF-at in the presence of 0.2% arabinose. The results indicated that MazF-at inhibits cell growth as a toxin of the TA system, and MazE-at neutralizes the MazF-at toxicity as an antitoxin of the TA system in the same operon.

### 3.2. The MazF-at Belongs to the MazF Family and E24 of MazF-at Is an Active Residue for Its Toxicity

**Figure 2:**
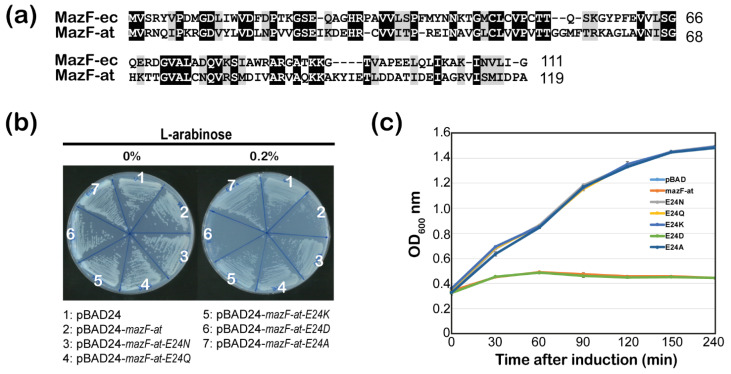
The identification of active residues in MazF-at for ribonuclease activity. (**a**) Sequence alignments between MazF-at and MazF-ec. Residues were shaded according to BLOSUM62 score. Dark- and grey- shaded residues represent identical and similar amino acid residues, respectively; (**b**) Toxicity of MazF-at mutants, MazF-at-E24N, -E24Q, -E24K, -E24D, and -E24A, on plates. The cells of *E. coli* BW25113 harboring each *mazE-at* mutant in pBAD24 were incubated on the M9 plate with or without 0.2% L-arabinose; (**c**) Effect of MazF-at mutants, MazF-at-E24N, -E24Q, -E24K, -E24D, and -E24A induction on cell growth. The culture of *E. coli* BW25113 harboring each *mazE-at* mutant in pBAD24 was grown until the OD_600nm_ of the culture reached ~0.4, followed by adding 0.2% L-arabinose at 0 min, and bacterial growth was measured for 6 h at OD_600_ nm. Error bars represent standard error of the mean (SEM).

The MazF-at is one of the MazF homologs in *A. tumefaciens*. MazF is reported in *E. coli* as an mRNA endoribonuclease that has the ability to cleave mRNA. To explore this possibility, we analyzed the global sequence alignment using the amino acid sequences of MazF-at and MazF-ec. The results showed that these two proteins have 33.3% identity and 45.5% similarity (Figure 2a). From a previous study on the tertiary protein structure of MazF-ec, a purified MazF-ec E24A mutant was used because it is impossible to purify wild-type MazF-ec from *E. coli* due to its toxicity. The E24A mutant is shown to have less toxicity than the non-mutated form, and the glutamic acid (E24) of MazF-ec has been determined as an active residue for the mRNA endoribonuclease activity [23]. In a recent study on the crystal structure of the E24 mutant, it is shown that the E24A mutant of MazF-ec affected substrate recognition [24,25]. Therefore, we created MazF-at mutants such as E24N, E24Q, E24K, E24D, and E24A in MazF-at and tested the cell growth on plates and in liquid medium with or without 0.2% L-arabinose. Glutamic acid was replaced by a different type of amino acids, such as polar uncharged side chain (N and Q), positive charged side chain (K), negative charged chain (D), or hydrophobic side chain (A). The results showed that the MazF-at mutants (E24D) have only preserved their toxicity, like wild-type MazF-at, and that the other mutants, MazF-at E24N, E24Q, E24K, and E24A, did not inhibit cell growth under 0.2 % arabinose (Figure 2b). In a liquid culture, the growth curve also indicated that MazF-at (E24D) had a cell growth arrest similar to that of wild-type MazF-at (Figure 2c). This might be because aspartic acid is a negatively charged amino acid, as glutamic acid. These results suggest that the glutamic acid (E24) amino acid of MazF-at is one of the active residues responsible for the toxicity of MazF-at and the MazF homolog, which is an endoribonuclease.

### 3.3. MazF-at Inhibits Protein Synthesis

**Figure 3:**
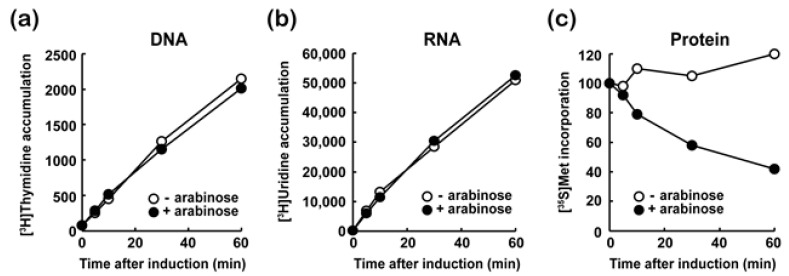
Effect of MazF-at induction on (**a**) DNA, (**b**) RNA, and (**c**) protein synthesis in vivo. The culture harboring pBAD24-*mazF-at* was grown until the OD_600nm_ of the culture reached 0.4, then MazF-at was overexpressed by induction with arabinose to a final concentration of 0.2%. Each 0.4 mL cell culture was sampled at 0, 5, 10, 30, and 60 min after the induction and was mixed with [^3^H]-thymidine (**a**), [^3^H]-uridine (**b**), or [^35^S]-methionine (**c**).

Toxins in TA systems have various targets, such as the essential cellular process of DNA, RNA, or protein synthesis to regulate cell growth according to environmental conditions. Therefore, to evaluate the effect of MazF-at on cell growth, we examined the effect of MazF-at induction with or without 0.2 % arabinose on DNA, RNA, and protein synthesis using [^3^H]-thymidine, [^3^H]-uridine, or [^35^S]-methionine in *E. coli* BW25113 cells, respectively. The incorporation of [^3^H]-thymidine (Figure 3a) or [^3^H]-uridine (Figure 3b) was not significantly affected upon MazF-at induction. However, MazF-at blocked the incorporation of [^35^S]-methionine into cellular proteins within 10 min of MazF-at induction (Figure 3c). This indicates that MazF-at inhibits protein synthesis, but not RNA and DNA synthesis.

### 3.4. MazF-at Is an Endoribonuclease

**Figure 4:**
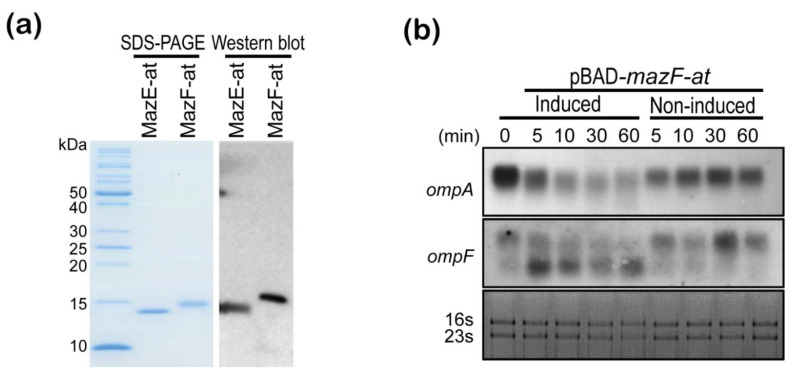
(**a**) Protein purification and Western blot assay using anti-his_6_ antibody of MazE-at and MazF-at. 1.5 ng of purified MazE-at and MazF-at proteins with N-terminal His_6_-tag were separated by 15% SDS-PAGE gel, and the gel was stained with Coomassie blue (left panel). Western blot analysis was performed on PVDF, and the blots were detected by anti-his_6_ antibody (right panel); (**b**) Effect of MazF-at on cellular mRNA stability using Northern blot assay. The total RNAs were extracted from *E. coli* BW25113 cells harboring pBAD24-*mazF-at* with (induced) or without 0.2% L-arabinose (non-induced) at 0, 5, 10, 30, and 60 min after the induction, and Northern blotting was performed using the *ompF* or *ompA* mRNA specific probes labeled with biotin-14-dCT.

Next, we purified MazE-at, MazF-at, and MazEF-at complex proteins using pET28-*mazE-at*, -*mazF-at*, and *–mazEF-at* with a His_6_-tag at the N-terminal end to confirm the activity of MazF-at in vitro. The molecular weight and purity of purified MazE-at, MazF-at, and MazEF-at were confirmed by SDS-PAGE and Western blot (Figure 4a). In addition, we evaluated whether MazF-at inhibited protein synthesis by decreasing cellular mRNA stability as MazF or YhaV does in *E. coli* using Northern blot. When MazF-at was overexpressed in the presence of 0.2% arabinose, the degradation of cellular mRNAs (*ompA, ompF*) was observed 5 min after induction (Figure 4b). These results suggest that MazF-at in the MazEF-at TA system has potential as an endoribonuclease in *A. tumefaciens*.

**Figure 5:**
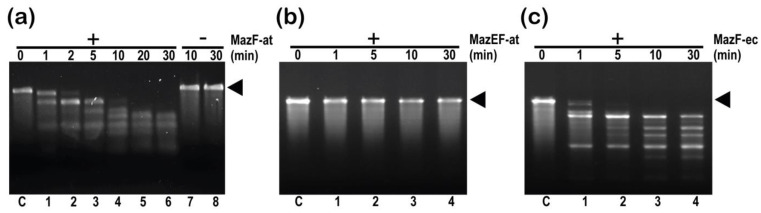
Endoribonuclease activity assay of (**a**) MazF-at, (**b**) MazEF-at complex, and (**c**) MazF-ec on MS2 phage RNA. 0.5 μM of MazF-at, MazEF-at, or MazF-ec protein was incubated with 0.8 μg/µL MS2 phage RNA, and the reaction was loaded on 1.5% agarose gel. The arrowheads indicate the full-length of MS2 phage RNA.

Next, to evaluate the in vitro endoribonuclease activity of purified MazF-at, the MazF-at or MazEF-at complex was incubated with MS2 phage RNA, and its cleavage patterns were compared with those of MazF-ec(E24A) (Figure 5c). MazF-at cleaved MS2 phage RNA from 1 min and completely digested the full-size MS2 phage RNA within 10 min (Figure 5a). Notably, MazEF-at did not cleave MS2 phage RNA even after 30 min of incubation (Figure 5b). The results indicated that MazF-at has in vitro endoribonuclease activity and its cognate antitoxin, MazE-at, can inhibit the activity of MazF-at (Figure 5).

### 3.5. Characterization of Purified MazE-at and MazF-at Proteins

**Figure 6:**
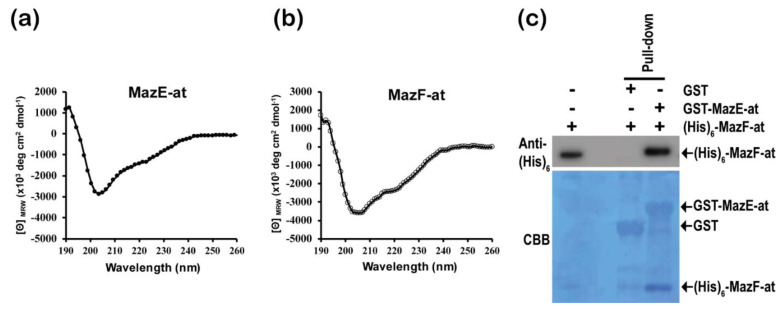
Circular dichroism (CD) analysis and the GST pull-down assay of MazE-at and MazF-at. The CD spectrum of MazE-at (**a**) and MazF-at (**b**) were measured from 190 to 260 nm wavelength (far-UV), and 0.25 mg/mL of purified protein was used; (**c**) Western blot (upper panel) and Coomassie blue staining (down panel) for GST pull-down assay of GST only, GST-MazE-at, and N-terminal His_6_ tag-MazF-at.

To investigate the secondary structure of MazE-at and MazF-at, we used far-UV circular dichroism (CD) with 0.25 mg/mL of purified proteins. The CD spectrum of MazE-at showed two negative peaks at approximately 208 and 222 nm that consist of α-helices (30.9%), β-sheets (31.5%), turns (19.7%), and random coils (18.0%) (Figure 6a). Moreover, MazF-at also has two negative peaks at approximately 208 and 222 nm, which consist of α-helices (28.9%), β-sheets (32.1%), turns (19.6%), and random coils (21.9%) (Figure 6b). The components of the secondary structures were calculated through CDNN [21]. These results suggest that the two proteins have similar secondary structure compositions. Normally, the antitoxin of type II TA systems has the ability to block the activity of its cognate toxins by forming a TA complex through protein-protein interactions. To confirm the in vitro interaction of the antitoxin MazE-at with MazF-at, we performed a pull-down assay using GST-MazE-at fusion protein. When MazF-at was incubated with GST or GST-MazE-at immobilized beads, GST-MazE-at only showed the band of MazF-at with His_6_-tag at the N-terminal end from the Western blot assay using anti-his_6_ antibody. This result showed that MazE-at directly interacts with MazF-at in vitro (Figure 6c).

## 4. Discussion

Here, we identified a MazEF TA system candidate in the *A. tumefaciens* genome and showed that MazF-at is a toxin and that the gene overlaps by one base with MazE-at, which functions as an antitoxin for MazF-at. The PemK family in the conserved domain database includes different toxins, such as MazF, Kid, PemK, ChpA, ChpB, and ChpAK in TA systems [26]. The Kis-Kid TA system is also known as the PemIK TA system. The Kis-Kid in plasmid R1 is known to encode the addiction modules to prevent the loss of plasmids through a mechanism known as post-segregational killing [7,25,26]. Recently, a MazEF homolog (MazF-dr) in *Deinococcus radiodurans* responds by an increase in ROS accumulation upon DNA damage stress [27]. Moreover, MazF in *Staphylococcus aureus* may contribute to reversible bacteria dormancy through activation of translation rescue, ribosome hibernation, the increase of cell wall thickness, and the decrease of cell division [28].

The amino acid alignment showed a high homology between MazF-ec from *E. coli* and MazF-at with 33.3% identity and 45.5% similarity. In addition, MazF-at has a high structural similarity with MazF-ec using a J-pred server (Figure S2 and Table S2) [29]. The active residue of MazF-ec was predicted to be the 24th glutamic acid because the toxicity of MazF-ec was reduced tenfold when the 24th glutamic acid was replaced by alanine (E24A) [23]. Based on this result, we changed the 24th glutamic acid of MazF-at (MazF-at-E24A) to alanine and confirmed that the E24 of MazF-at is an active residue for its toxicity using site-directed mutagenesis in a manner similar to MazF-ec. However, the MazF-homolog from *Bacillus subtilis* has its catalytic key residues at R25 and T48 from the crystal structure and site-directed mutagenesis assay. Therefore, it is necessary to study whether there are more active residues of MazF-at besides E24. In addition, it is not known whether they are involved in binding to target RNA, endoribonuclease activity, or both. In order to prove this, further studies are required to determine the tertiary structure of MazF-at.

Previous studies have shown that MazF homologs in other bacteria have mRNA interferase activity to cleave mRNA. Here, we evaluated whether MazF-at also has endoribonuclease activity, as MazF does in *E. coli*. First, we examined whether MazF-at affects cell growth by inhibiting important cellular processes and found that MazF-at is an inhibitor for the synthesis of all cellular proteins, but not DNA and RNA synthesis. In addition, to evaluate whether MazF-at inhibits protein synthesis by decreasing cellular mRNA stability, MazF-at was overexpressed in vivo, and we found that the cellular mRNAs (*ompA, ompF*) were degraded following specific time intervals. To confirm that this RNA degradation was due to MazF-at, we purified MazE-at, MazF-at, MazEF-at complex, and MazF-E24A, and the decrease in RNA stability after the incubation of purified MazF-at with MS2 phage RNA was evaluated in vitro. Ideally, all of these experiments should be done in its original host, *A. tumefaciens*. However, it has been proven to have toxicity with overexpressed MazF-at in *A. tumefaciens*, and it is difficult to obtain a sufficient amount of proteins from *A. tumefaciens* for in vitro studies, because its doubling time is 2.5–4 hrs. For this reason, this study was conducted using *E. coli*, although MazF-at may have different physiological function in *A. tumefaciens*. From these results, we showed that MazF-at has a ribosome-independent endoribonuclease activity because MazF-at showed endoribonuclease activity without ribosomes in vitro. Usually, ribosome-independent mRNA interferases recognize and cleave specific RNA sequences and specific lengths. The MqsR homolog from *E. coli* specifically cleaves intracellular mRNAs at a GCU sequence [30]. Recently, it was reported that an MazF homolog from *E. coli* recognized about seven nucleotide regions with extended recognition specificity for ACA and its flaking sequences [31]. The protein purification of MazF-ec or MqsR has not been possible without the modification of the protein, but an MazF homolog (MazF-cd) from *Clostridium difficile*, which cleaves 5bp UACAU sequences of mRNA, was successfully purified [32]. This might be because of the different level of toxicity of those toxins, but what makes this difference in toxicity is still unknown. Furthermore, the global analysis of the cleavage site of MazF showed that *E. coli* MazF cleaved rRNA precursors and ribosomal protein transcripts, which may inhibit ribosome biogenesis [31,33]. However, AtYoeB showed ribosome-independent non-specific nuclease activity cleaving both RNA and DNA [19]. Therefore, further study is needed to identify whether MazF-at has specific recognition sites and the length of the cleavage site.

The antitoxin of the type II TA system inhibits toxin activity by forming a TA complex through protein-protein binding. To confirm whether MazE-at also binds to MazF-at through direct protein-protein interaction, we examined the pull-down assay, which showed the direct binding between MazE-at and MazF-at. Moreover, we evaluated the secondary structure of MazE-at and MazF-at. The two proteins have typical helix structures. Usually, although many antitoxins in type II TA systems have an unstructured region of free antitoxin, the disordered domain of antitoxin is highly susceptible to stress-induced proteases [34]. Interestingly, MazE-at has a stable secondary structure, unlike other antitoxins. Almost all antitoxins block the toxicity of their cognate toxins by direct binding to their active sites. MazE-at antitoxin binding to MazF-at may induce a conformational change to inhibit the activity of MazF-at.

In this study, we showed that the MazEF-at toxin-antitoxin system is located in the *A. tumefaciens* chromosome and one of the MazF homologs. MazF-at in the MazEF TA system has endoribonuclease nuclease activity, and its cognate antitoxin MazE-at may neutralize the toxicity of MazF-at. This might help us to find the function of TA systems in *A. tumefaciens* and to develop its application.

## Figures and Tables

**Table 1:** Strains and plasmids used in this study.

Strain and Plasmid	Description	Source
Strains		
A. tumefaciens EHA105	Genomic DNA for cloning	Lab stock
*E. coli* DH5α	General cloning host	Lab stock
*E. coli* BL21(DE3)	General protein expression host	Lab stock
*E. coli* BW25113	Wild type	Lab stock
Plasmids		
pBAD24	Gene expression vector, P_BAD_, amp	Lab stock
pBAD24-*mazE-at*	*mazE* in pBAD24	This study
pBAD24- *mazF-at*	*mazF* in pBAD24	This study
pBAD24- *mazEF-at*	*mazEF* in pBAD24	This study
pBAD24- *mazF-at* (E24N)	Mutant MazF of E24N in pBAD24	This study
pBAD24- *mazF-at* (E24Q)	Mutant MazF of E24Q in pBAD24	This study
pBAD24- *mazF-at* (E24K)	Mutant MazF of E24K in pBAD24	This study
pBAD24- *mazF-at* (E24D)	Mutant MazF of E24D in pBAD24	This study
pBAD24- *mazF-at* (E24A)	Mutant MazF of E24A in pBAD24	This study
pET28a	Protein expression vector, P_IPTG_, kan	Lab stock
pET28- *mazE-at*	*mazE* in pET28a	This study
pET28- *mazF-at*	*mazF* in pET28a	This study
pET28- *mazEF-at*	*mazEF* in pET28a	This study
pGEX6p-1	Protein expression vector, P_IPTG_, amp	Lab stock
pGEX6p-1-*mazE-at*	*mazE* in pGEX6p-1	This study

**Table 2:** Primers used in this study.

Primer	Sequence	Source
A-mazE-F	5′-TATAGGATCCATGACCGTGACCACGA-3′	This study
A-mazE-R	5′-TATAAAGCTTTCACAACGCTTCTTTG-3′	This study
T-mazF-F	5′-TATACATATGGTCCGCAACCAGAT-3′	This study
T-mazF-R	5′-TATACTCGAGTCAAGCTGGATCGATCAT-3′	This study
TA-mazEF-F	5′-TATAGGATCCATGACCGTGACCACGA-3′	This study
TA-mazEF-R	5′-TATACTCGAGTCAAGCTGGATCGATCAT-3′	This study
Pull-mazE-F	5′-TATAGGATCCATGACCGTGACCACGA-3′	This study
Pull-mazE-R	5′-TATAGCGGCCGCTCACAACGCTTCTTTGC-3′	This study
Mu-MazF-E24N-F	5′-GTCGTAGGCAGCAACATCAAGGACGAA-3′	This study
Mu-MazF-E24N-R	5′-TTCGTCCTTGATGTTGCTGCCTACGAC-3′	This study
Mu-MazF-E24Q-F	5′-GTCGTAGGCAGCCAAATCAAGGACGAA-3′	This study
Mu-MazF-E24Q-R	5′-TTCGTCCTTGATTTGGCTGCCTACGAC-3′	This study
Mu-MazF-E24K-F	5′-GTCGTAGGCAGCAAAATCAAGGACGAA-3′	This study
Mu-MazF-E24K-R	5′-TTCGTCCTTGATTTCGCTGCCTACGAC-3′	This study
Mu-MazF-E24D-F	5′-GTCGTAGGCAGCGACATCAAGGACGAA-3′	This study
Mu-MazF-E24D-R	5′-TTCGTCCTTGATGTCGCTGCCTACGAC-3′	This study
Mu-MazF-E24A-F	5′-GTCGTAGGCAGCGCAATCAAGGACGAA-3′	This study
Mu-MazF-E24A-R	5′-TTCGTCCTTGATTGCGCTGCCTACGAC-3′	This study
ompA1-F	5′-GGTGCATACAAAGCTCAGGG-3′	[6]
ompA1-R	5′-GTGACTGCGTACTCAACACC-3′	[6]
ompF1-F	5′-CTTTGGTCTGGTTGATGGCC-3′	[6]
ompF1-R	5′-ACTTCAGACCAGTAGCCCAC-3′	[6]

## Data Availability

Data are contained within the article or Supplementary Materials.

## Supplementary Materials

The following are available online at https://www.mdpi.com/article/10.3390/microorganisms9051107/s1, Figure S1: Effect of 13 candidates’ induction for TA systems identified in *A. tumefaciens* C58 on *E. coli* BW25113 cell growth. The pBAD24, pBAD24-*aut0940*, -*aut1078*, -*aut1311*, -*aut1628*, -*aut8169*, -*aut2033*, -*aut2326*, -*aut2017*, -*aut0246*, -*aut0674*, -*aut0849*, -*aut0934*, and –*aut1004* were transformed into *E. coli* BW25113. To conduct the cell toxicity assay, transformed cells were grown on M9 agar plates with or without 0.2% L-arabinose at 37 °C for 20 h; Figure S2: Secondary structure prediction of MazF-at using Jpred 4, a protein secondary structure prediction server. Lupas_21, Lupas_14, and Lupas_28 represent coiled-coil predictions for the sequence. JNetPRED and JNetCONF represent the consensus prediction and the confidence estimate for the prediction (high values mean high confidence), respectively. JNETSOL25, JNETSOL5, and JNETSOL0 are the binary predictions of 25%, 5%, and 0% solvent accessibility, respectively. JNetHMM and JNETPSSM show the HMM-profile-based prediction and PSSM-based prediction, respectively. JNETJURY indicates the locations to rationalize the significantly different prediction residues with an asterisk; Table S1: TA system candidates from A. tumefaciens C58 genome using the toxin-antitoxin database (TADB 2.0); Table S2: High structural similarity homologs of MazF-at using a J-pred server.
