# Supplementary material for: Functional Characterization of the mazEF Toxin-Antitoxin System in the Pathogenic Bacterium Agrobacterium tumefaciens
Source: Microorganisms. 2021 May 20;9(5):1107. doi: 10.3390/microorganisms9051107 (PMC8160871; doi:10.3390/microorganisms9051107)

Arabinose concentration

0% 0.2%

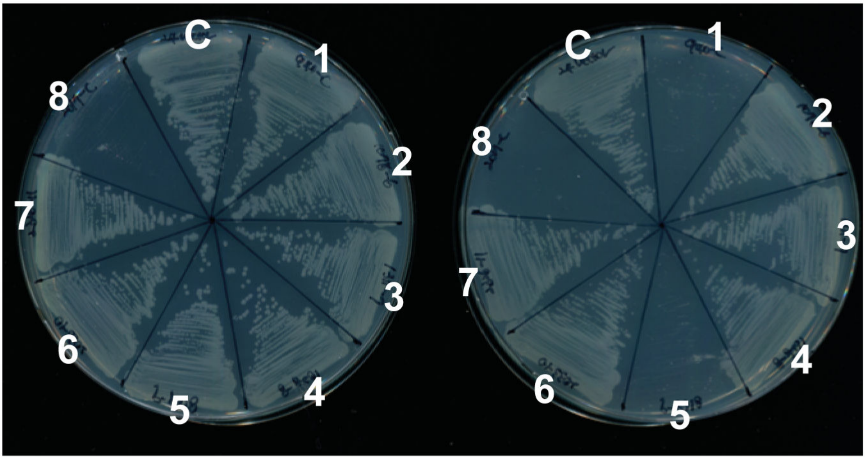

C: pET24
1: pET24-*atu0940*
2: pET24-*atu1078*
3: pET24-*atu1311*
4: pET24-*atu1628*
5: pET24-*atu8169*
6: pET24-*atu2033*
7: pET24-*atu2326*
8: pET24-*atu2017*
9: pET24-*atu0246*
10: pET24-*atu0674*
11: pET24-*atu0849*
12: pET24-*atu0934*
13: pET24-*atu1004*

Arabinose concentration

0% 0.2%

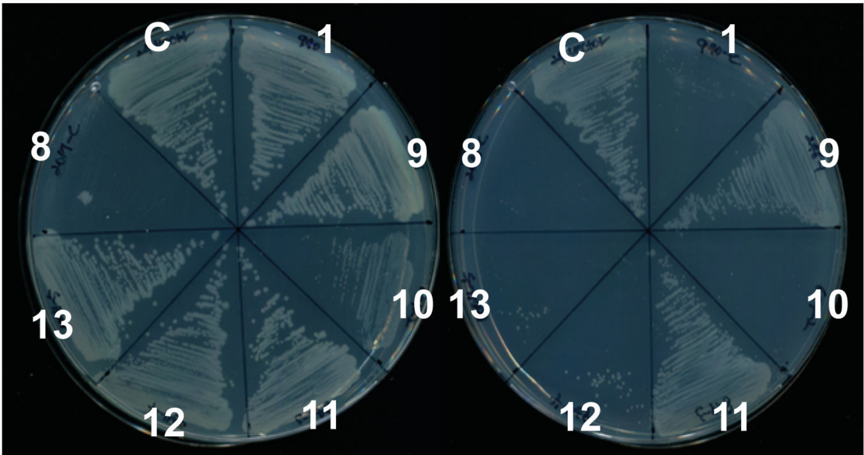

Supplement: Supplementary file 1 [file microorganisms-09-01107-s001.zip › Figure S1.pdf]
