# Supplementary material for: Functional Characterization of the mazEF Toxin-Antitoxin System in the Pathogenic Bacterium Agrobacterium tumefaciens
Source: Microorganisms. 2021 May 20;9(5):1107. doi: 10.3390/microorganisms9051107 (PMC8160871; doi:10.3390/microorganisms9051107)
Supplement: Supplementary file 1 [file microorganisms-09-01107-s001.zip › Table S1.pdf]

Table S1. TA system candidates from *A. tumefaciens* C58 genome.

No	Type	Toxin	Antitoxin	Location	Classification(family/domain)
1	2	Atu0940	Atu0939	928967..929592	mazEF/AbrB-MazF
2	2	Atu1078	Atu1079	1070711..1071287	RelBE or parDE/RHH-RelE
3	2	Atu1311	Atu1312	1304040..1305011	-/RHH-GNAT
4	2	Atu1628	Atu1629	1614348..1615015	-/ArsR-COG3832
5	2	Atu8169	Atu2028	1988551..1989404	-/ArsR-COG3832
6	2	Atu2033	Atu2034	1992395..1993289	-/ArsR-COG3832
7	2	Atu2326	Atu2325	2301360..2302012	-HEPN-MNT
8	2	Atu2017	Atu2018	1979333..1979853	RelBE or parDE/RHH-RelE
9	2	Atu0246	Atu0245	243980..245097	-/Xre-GNAT
10	2	Atu0674	Atu0675	670257..670737	relBE/RHH-RelE
11	2	Atu0849	Atu0848	849385..850489	-/COG5642-COG5654
12	2	Atu0934	Atu0935	924141..924748	relBE/RHH-RelE
13	2	Atu1004	Atu1005	1001210..1001826	vapBA/RHH-PIN
