# Supplementary material for: Functional Characterization of the mazEF Toxin-Antitoxin System in the Pathogenic Bacterium Agrobacterium tumefaciens
Source: Microorganisms. 2021 May 20;9(5):1107. doi: 10.3390/microorganisms9051107 (PMC8160871; doi:10.3390/microorganisms9051107)
Supplement: Supplementary file 1 [file microorganisms-09-01107-s001.zip › Table S2.pdf]

Table S2. High structural similarity homologs of MazF-at using J-pred server.

PDB	Chain	Description	Blast E-value
2c06	B	KID TOXIN PROTEIN	1.00E-13
2c06	A	KID TOXIN PROTEIN	1.00E-13
1m1f	B	Kid toxin protein	1.00E-13
1m1f	A	Kid toxin protein	1.00E-13
5cr2	C	Endoribonuclease MazF	8.00E-08
5cr2	B	Endoribonuclease MazF	8.00E-08
5cr2	A	Endoribonuclease MazF	8.00E-08
5co7	F	Endoribonuclease MazF	8.00E-08
5co7	E	Endoribonuclease MazF	8.00E-08
5co7	D	Endoribonuclease MazF	8.00E-08
5co7	C	Endoribonuclease MazF	8.00E-08
5co7	B	Endoribonuclease MazF	8.00E-08
5co7	A	Endoribonuclease MazF	8.00E-08
5ckb	B	Endoribonuclease MazF	8.00E-08
5ckb	A	Endoribonuclease MazF	8.00E-08
5ck9	B	Endoribonuclease MazF	8.00E-08
5ck9	A	Endoribonuclease MazF	8.00E-08
3nfc	F	PemK-like protein 1	8.00E-08
3nfc	E	PemK-like protein 1	8.00E-08
3nfc	D	PemK-like protein 1	8.00E-08
3nfc	C	PemK-like protein 1	8.00E-08
3nfc	B	PemK-like protein 1	8.00E-08
3nfc	A	PemK-like protein 1	8.00E-08
1ub4	B	MazF protein	3.00E-07
1ub4	A	MazF protein	3.00E-07
5cqy	B	Endoribonuclease MazF	4.00E-07
5cqy	A	Endoribonuclease MazF	4.00E-07
5cqx	B	Endoribonuclease MazF	4.00E-07
5cqx	A	Endoribonuclease MazF	4.00E-07
5ckh	B	Endoribonuclease MazF	4.00E-07
5ckh	A	Endoribonuclease MazF	4.00E-07
5ckf	B	Endoribonuclease MazF	4.00E-07
5ckf	A	Endoribonuclease MazF	4.00E-07
5cke	B	Endoribonuclease MazF	4.00E-07
5cke	A	Endoribonuclease MazF	4.00E-07
5ckd	B	Endoribonuclease MazF	4.00E-07
5ckd	A	Endoribonuclease MazF	4.00E-07
7bye	F	Uncharacterized protein	3.00E-06
7bye	E	Uncharacterized protein	3.00E-06
7bye	C	Uncharacterized protein	3.00E-06
7bye	B	Uncharacterized protein	3.00E-06
